# Cognitive Rehabilitation for Attention and Memory in people with Multiple Sclerosis: study protocol for a randomised controlled trial (CRAMMS)

**DOI:** 10.1186/s13063-015-1016-3

**Published:** 2015-12-08

**Authors:** Nadina B. Lincoln, Roshan das Nair, Lucy Bradshaw, Cris S. Constantinescu, Avril E. R. Drummond, Alexandra Erven, Amy L. Evans, Deborah Fitzsimmons, Alan A. Montgomery, Miriam Morgan

**Affiliations:** Division of Rehabilitation and Ageing, School of Medicine B127a Medical School Queens Medical Centre, Nottingham, NG7 2UH UK; Department of Clinical Psychology & Neuropsychology, Nottingham University Hospitals NHS Trust, Nottingham, NG7 2UH UK; Nottingham Clinical Trials Unit, C floor South Block, Queens Medical Centre, Nottingham, NG7 2UH UK; Department of Clinical Neurology, South Block, Queens Medical Centre, University of Nottingham, Nottingham, NG7 2UH UK; School of Health Sciences, A Floor, South Block, Queens Medical Centre, University of Nottingham, Nottingham, NG7 2HA UK; Swansea Centre for Health Economics, College of Human and Health Sciences, Singleton Campus, Swansea University, Swansea, SA2 8PP UK

**Keywords:** Multiple sclerosis, Attention, Memory, Cognitive rehabilitation, Randomised controlled trial, Cost-effectiveness

## Abstract

**Background:**

People with multiple sclerosis have problems with memory and attention. Cognitive rehabilitation is a structured set of therapeutic activities designed to retrain an individual’s memory and other cognitive functions. Cognitive rehabilitation may be provided to teach people strategies to cope with these problems, in order to reduce the impact on everyday life. The effectiveness of cognitive rehabilitation for people with multiple sclerosis has not been established.

**Methods:**

This is a multi-centre, randomised controlled trial investigating the clinical and cost-effectiveness of a group-based cognitive rehabilitation programme for attention and memory problems for people with multiple sclerosis. Four hundred people with multiple sclerosis will be randomised from at least four centres. Participants will be eligible if they have memory problems, are 18 to 69 years of age, are able to travel to attend group sessions and give informed consent. Participants will be randomised in a ratio of 6:5 to the group rehabilitation intervention plus usual care or usual care alone. Intervention groups will receive 10 weekly sessions of a manualised cognitive rehabilitation programme. The intervention will include both restitution strategies to retrain impaired attention and memory functions and compensation strategies to enable participants to cope with their cognitive problems.

All participants will receive a follow-up questionnaire and an assessment by a research assistant at 6 and 12 months after randomisation. The primary outcome is the Multiple Sclerosis Impact Scale (MSIS) Psychological subscale at 12 months. Secondary outcomes include the Everyday Memory Questionnaire, General Health Questionnaire-30, EQ-5D and a service use questionnaire from participants, and the Everyday Memory Questionnaire-relative version and Carer Strain Index from a relative or friend. The primary analysis will be based on intention to treat. A mixed-model regression analysis of the MSIS Psychological subscale at 12 months will be used to estimate the effect of the group cognitive rehabilitation programme.

**Discussion:**

The study will provide evidence regarding the clinical and cost-effectiveness of a group-based cognitive rehabilitation programme for attention and memory problems in people with multiple sclerosis.

**Trial registration:**

ISRCTN09697576. Registered 14 August 2014.

## Background

Cognitive rehabilitation is a structured set of therapeutic activities designed to retrain an individual’s memory and other cognitive functions. A narrative review [1] reported that cognitive rehabilitation was beneficial for treating cognitive deficits following brain damage. There are recommendations for the provision of cognitive rehabilitation for people with multiple sclerosis (MS) in the European Federation of Neurological Societies Guidelines on cognitive rehabilitation [2] and National Service Framework for Long term Conditions [3]. Some randomised controlled trials (RCTs) have demonstrated the effectiveness of cognitive rehabilitation in people with MS [4–8] but most evidence comes from single case experimental design studies, non-RCTs, and small pilot RCTs [9]. Systematic reviews on cognitive rehabilitation have not found evidence to support or refute the effectiveness of cognitive rehabilitation for people with MS [10–13]. However, the narrative review by O’Brien et al. [9] found low-level evidence for positive effects of neuropsychological rehabilitation in MS and suggested that more high quality trials were needed.

Two small scale pilot RCTs used similar cognitive rehabilitation programmes. The ReMIND trial [14] (n = 72) evaluated the effectiveness of group memory rehabilitation programmes in patients with memory problems, many of whom had MS (n = 39). Participants were randomly allocated to one of three programmes: compensation strategy training, restitution, or a self-help control. Both quantitative and qualitative data from the study [14, 15] indicated the interventions were worthy of further evaluation. The ReMIND-MS trial [16] was a modified version of the cognitive rehabilitation group intervention, combining restitution and compensation strategies, compared with a usual care control with people with MS (n = 48). The results showed a significant effect on mood, favouring the intervention group. These two pilot RCTs have informed the sample size calculations and assessment and treatment methods for this present trial.

This present trial has been designed to assess the clinical and cost-effectiveness of a group cognitive rehabilitation programme, on the basis of recent research suggestions [17], Cochrane reviews [11, 12], our own pilot studies [14, 16], current clinical guidelines [2, 3] and clinical practice in the UK.

## Methods

### Trial objectives

The primary objective is to determine whether attending a group cognitive rehabilitation programme (the intervention), in addition to usual care, is associated with reduced psychological impact of MS on quality of life, as measured on the MS Impact Scale (MSIS) Psychological Subscale (MSIS-Psy) [18] when compared to usual care alone (control). The secondary objectives are to assess cost-effectiveness of the intervention, and whether the intervention is associated with improvements in participants’ attention and memory abilities, self-reported attention, memory problems in daily life, mood, fatigue, employment status and carer strain.

### Trial design

This is a multi-centre, parallel group, RCT.

### Ethical approval

Ethical approval was granted by NRES Committee West Midlands on 1 September 2014 (reference 14/WM/1083).

### Site and participant recruitment

The study will be conducted in at least four centres in the UK.

Participants will be identified through National Health Service hospitals, rehabilitation centres and charities (e.g. MS Society branches). A letter will be sent to individuals, identified as potential participants, by a member of the clinical team, which will include a participant information sheet, a consent form and a pre-paid reply envelope. Self-referral will also be possible for those who access public facing information, on the study website, newsletters and posters. Recruitment will take place over 2 years.

### Informed consent

Written informed consent will be obtained by an assistant psychologist. Participants will be informed that their participation is entirely voluntary and they are free to withdraw at any time; in the event of their withdrawal, any data collected up until that point would be kept by the research team. Participants will be asked whether they consent to a follow-up interview to assess treatment acceptability and will be informed that, if allocated to the intervention group, sessions may be video recorded to ensure treatment fidelity. A letter will be sent to the General Practitioners of consenting participants informing them of their patients’ involvement in the trial.

### Inclusion criteria

People with MS are eligible for the trial if they are aged 18 to 69 years, have relapsing remitting or progressive MS, diagnosed at least 3 months prior to the baseline assessment, report having cognitive problems as determined by a cut-off score >27 on the patient version of the Multiple Sclerosis Neuropsychological Screening Questionnaire (MSNQ) [19], have cognitive deficits, defined as performance more than one standard deviation below the mean of healthy controls corrected for age and education [20] on the Brief Repeatable Battery of Neuropsychological Tests (BRBN) [21], are able to travel to one of the centres to attend group sessions, are able to speak English sufficiently to complete the cognitive assessments and take part in group sessions, and give informed consent.

### Exclusion criteria

Potential participants will be excluded if they have vision or hearing problems, such that they are unable to complete the cognitive assessments, have concurrent severe medical or psychiatric conditions, which would prevent them from engaging in treatment, or are involved in other psychological intervention trials.

### Initial screening assessment

At the first appointment, the assistant psychologist will explain the study and make clear that the initial screening assessments are required to check that the participant meets the inclusion criteria and to obtain some baseline data for those who are eligible. Demographic information recorded will include gender, date of birth, ethnicity, years of education, living arrangements, marital status and employment status.

The following assessments will be conducted at the initial screening:

- Multiple Sclerosis Neuropsychological Screening Questionnaire (MSNQ) [19]
- The Brief Repeatable Battery of Neuropsychological Tests (BRBN) [21]
- Guy’s Neurological Disability Scale [22]

The results from the MSNQ and BRBN will be used to assess whether the participant meets the inclusion criteria. Following screening, participants will be informed whether they meet the study criteria. Those who do not will be notified and thanked for their interest in the study.

Those who meet the inclusion criteria will be given questionnaires to complete in their own time. These will be collected at the baseline assessment visit:

- MS Impact scale version 2 (MSIS) [18]
- Everyday Memory Questionnaire (EMQ) patient version [23]
- General Health Questionnaire 30 item (GHQ30) [24]
- Fatigue Severity Scale (FSS) [25]

Participants will be sent an information sheet for a friend or relative with an EMQ relative version to be completed and returned.

At the baseline visit the following assessments will be conducted by the assistant psychologist:

- Euroquol five dimensions five levels (EQ-5D-5L) [26]
- Use of Health and Social Services Questionnaire, which also includes medication and medication changes.
- Doors and People [27]
- Trail Making Test [28]

The assistant psychologist will check the participant’s availability to attend groups on certain days, should they be randomised to receive the intervention. Participants will only be randomised if they can attend on the days that groups are scheduled. Those unable to attend on scheduled days will be held in reserve until such time that a new group, matching their availability, is formed. During the period participants are waiting for a sufficient number of other participants to be included in a group, the assistant psychologist will remain in regular contact to keep individuals aware of likely timescales.

### Participant outcome assessments

Outcomes will be assessed at 6 and 12 months after randomisation to assess immediate and long-term effects of the intervention. The primary follow-up is 12 months after randomisation. The NHS number will be supplied to the Medical Research Information Service to allow a mortality check prior to contact for follow-up.

The primary outcome is the psychological impact of MS, measured using the MSIS-Psy [18].

Secondary outcomes are:

- Memory problems in everyday life, as measured using the EMQ patient and relative versions [23]
- Mood, as measured using the GHQ30 [24]
- Fatigue, as measured using the FSS [25]
- Quality of Life, as measured using the EQ-5D-5L [26]
- Attention and memory abilities, as measured by a cognitive test battery
  - ○ BRBN [21]
  - ○ Doors and People [27]
  - ○ Trail Making Test [28]
- Physical impact of MS, as measured using the MSIS Physical Subscale (MSIS-Phys) [18]
- Cost-effectiveness, with costs measured by the Use of Health and Social Services Questionnaire compared with the primary and secondary outcomes, including a cost per quality-adjusted life year analysis using the EQ-5D-5L [26]
- Employment status, as measured as part of the Use of Health and Social Services Questionnaire
- Carer strain, as measured using the Modified Carer Strain Index [29]

In addition, the level of disability, as measured by the Guys Neurological Disability Scale (GNDS) [22] and number of reported MS relapses in the previous 6 months, will be recorded.

Participants will receive a questionnaire pack, which includes the MSIS, EMQ, GHQ30, FSS and GNDS, to complete in their own time. They may request help in completing these questionnaires if necessary. A research assistant (RA), who is unaware of the group allocation, will check whether the questionnaires have been completed prior to the assessment visit. If they have not, the RA will ask the participant to complete them during the visit and will also conduct the cognitive assessments, Use of Health and Social Services Questionnaire and the EQ-5D-5L [26].

### Minimisation of bias

Steps will be undertaken to reduce the risk of bias in this trial. Allocation will be randomly assigned and concealed using an automated web-based system operated by Nottingham Clinical Trials Unit (NCTU). There is a single primary outcome (MSIS-Psy) [18] and all outcomes specified in the protocol will be analysed and reported. While the primary outcome and some secondary outcomes are self-reported and therefore not blinded, the cognitive tests will be assessed by a researcher who will be blinded to treatment allocation. Collection of outcome data will be attempted from every randomised participant not known to have died at the time of follow-up and who has not withdrawn consent, regardless of adherence to allocated treatment. It is anticipated that there will be some non-collection of primary outcome data, and while the primary intention-to-treat analyses will be without imputation of missing data, sensitivity analyses will investigate various assumptions about the missing data.

The participants and assistant psychologists will not be blind to the allocated treatment. To prevent unblinding, the RA will request participants completing outcome assessments not to discuss any aspect of being involved with the study. The RA will also be required to guess the treatment allocation for each participant and this will be compared later to the actual allocation, to determine the degree of unblinding.

### Randomisation

Participants will be individually randomised to intervention or control on a 6:5 ratio to allow for clustering in the intervention arm. Allocation will be stratified by recruitment site, and minimised by MS type (relapsing–remitting or progressive) and gender. Randomisation will take place once there are 9–11 individuals who have consented and who are able to attend the same therapy group (location, day of the week and time of day) should they be randomised to receive it. The allocation algorithm will be created by the NCTU in accordance with their standard operating procedure and held on a secure server. Assistant psychologists at each site will use a remote, internet-based randomisation system to obtain treatment allocations for each participant. Access to the sequence will be confined to the NCTU Information Technology Manager. The sequence of treatment allocations will be concealed from the study statistician until all participants have been allocated, and recruitment, data collection and all other study-related assessments are complete.

### Duration of participant participation

**Fig. 1:**
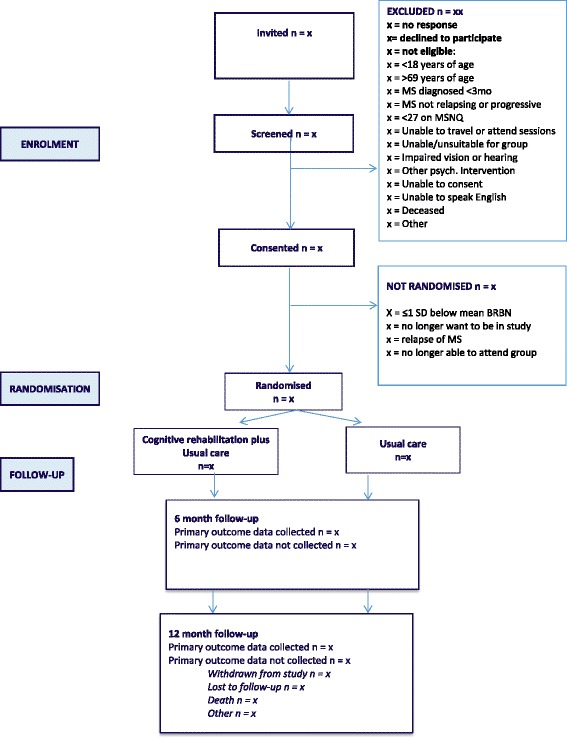
CONSORT diagram. *BRBN* Brief Repeatable Battery of Neuropsychological Tests, *MS* Multiple sclerosis, *MSNQ* Multiple Sclerosis Neuropsychological Screening Questionnaire

Figure 1 shows the expected progress of the study. Participants are in the study for approximately 14 months from the initial screening assessment (12 months from randomisation). Participants will leave the study when they have completed the 12 month follow-up.

### Cognitive rehabilitation

Cognitive rehabilitation is a structured set of therapeutic activities designed to improve cognitive function and to reduce the impact of cognitive impairment on daily life. The emphasis of the intervention will be on identifying the most appropriate strategies to help individuals overcome their cognitive problems and in providing participants with a range of techniques, which they can use and adapt according to their needs.

Each rehabilitation group will be led by an assistant psychologist under the supervision of a clinical psychologist. Assistant psychologists will be trained centrally on all study-related procedures, including the delivery of the intervention, to ensure consistency across sites and adherence to the protocol. Each group will consist of four to six participants. Participants will receive 10 group memory rehabilitation sessions (1.5 hours long, including a break, once a week for 10 weeks). The content of sessions will be as defined in a treatment manual, which was developed and tested in the previous study [16]. The intervention will include restitution strategies to retrain memory functions, attention retraining and strategies to improve encoding and retrieval. Compensation strategies will also be taught, including internal mnemonics (such as chunking, use of first letter cues, rhymes), use of external devices (such as diaries, mobile phones, calendars) and ways of coping with memory problems. The programme will be tailored to each participant’s cognitive status, depending on the impairments identified during the baseline assessment, while maintaining a systematic approach to working on attention and memory functions. Each session will end with the setting of homework assignments to help participants practice the strategies learnt in the group sessions in their daily life. These will be reviewed at the following session. Carers and family members will be invited to attend the last session, if participants agree, which serves as a summary of the previous sessions.

### Control group (usual care)

Participants will receive their usual clinical care. In the standard NHS care pathway, people with MS with cognitive problems may get general advice from MS nurses and occupational therapists on how to manage any cognitive difficulties. There are information sheets available on web pages of MS charities which include suggestions for coping. However, usual care does not normally include any specific intervention for cognitive problems or cognitive rehabilitation.

All other clinical services will be available as usual for both groups. This may include referral to employment rehabilitation services, self-help groups or support from specialist charities, such as the MS Society. Any additional input (including psychological or medical interventions) participants receive during the study will be recorded from the Use of Health and Social Services questionnaire.

### Compliance with interventions

The assistant psychologist will record whether participants attend each of the treatment sessions and the reasons why any sessions are not attended, if known.

To ensure the fidelity of the intervention, at least twenty sessions will be video recorded. Sessions will be purposively pre-selected for recording in order to include sessions from the beginning, middle and end of the 10-week course and recordings will be made across the intervention period. Practices for video recording will draw upon guidance on minimising intrusiveness of the recording [30, 31]. Methods used in previous work will be used to analyse the content of training within rehabilitation contexts [32, 33]. Two independent assessors will separately analyse the video recordings using a customised score sheet to capture a variety of key elements spanning all aspects of the intervention. Assessors will code these factors as present or absent over a series of time intervals. This method has successfully been used in the pilot study to determine treatment fidelity without disrupting the group sessions [33].

### Sample size and justification

Our sample size estimate is based on analysis of the MSIS-Psy [18] at 12 months post-randomisation. A clinically meaningful effect using this outcome is probably in the range 3–3.5. In the pilot study, the 95 % confidence interval for the difference between intervention and usual care was −1 to +8, indicating that the intervention has the potential to have an effect that is regarded as clinically worthwhile. The common standard deviation in the pilot study was 7.5 (using version 1 of the MSIS-Psy scored 9 to 45). However, we expect the standard deviation will be higher in the present trial as the sample for the pilot study were all recruited from a single outpatient rehabilitation unit, whereas this proposed sample will include people who have been recruited from multiple settings.

Based on a two-sample test, 143 participants per arm are required for analysis in order to detect a difference of 3 points on the MSIS-Psy, assuming a standard deviation of 9 (effect size 0.33), with 80 % power, and 5 % two-sided alpha. However, a clustering effect may occur in the intervention arm due to the intervention being delivered in groups. We estimate this clustering effect to be 0.1 [13]. Design and analysis issues in partially clustered clinical trials have been reported [34]. Based on an average cluster size of five evaluable participants (those providing primary outcome data at 12 months after randomisation), and an intracluster correlation coefficient (ICC) of 0.1 in the intervention arm, a total of 336 evaluable patients would provide 80 % power to detect such a difference (184 to intervention and 154 to usual care). Additionally, the optimal allocation ratio depends on the cluster size and the ICC. In this case, we will allocate participants in a ratio of 6:5 in favour of the intervention arm.

Data from the pilot study suggested non-collection of primary outcome data in 8 % of participants. However because of the wider recruitment strategy and from evidence of recruitment to other related studies [35, 36], we estimate it will be 15 %. We will therefore aim to randomise a total of 400 participants (216 to intervention and 184 to usual care).

Version 2 of the MSIS-Psy will be used in this study with scores ranging between 9 and 36. The standard deviation of the MSIS-Psy version 2 in the UK South West Impact of Multiple Sclerosis cohort was 6.4 [37]. If the standard deviation in this study is between 6 and 9, differences of between 2 and 3 points on version 2 of the MSIS-Psy will be detectable based on the effect size specified above, with assumed similar clinical importance as for version 1.

### Statistical analysis

The analysis and presentation of the trial will be in accordance with CONSORT guidelines [38], with the primary between-group comparisons based on analysing participants as randomised without imputation of missing data. A full analysis plan will be developed prior to completion of data collection and discussed and agreed with the Trial Steering Committee and Data Monitoring Committee.

Descriptive statistics of demographic and clinical measures at baseline will be used to examine balance between those randomised to intervention and control. The primary analysis will employ a mixed effects linear regression model of the MSIS-Psy outcome at 12 months adjusted for baseline value and stratification/minimisation variables, and taking appropriate account of clustering by therapy group. Distributions of raw outcome scores and regression model residuals will be examined and the data suitably transformed or a non-parametric analysis employed if necessary. For a parametric analysis, the comparison will be presented as an adjusted difference in mean MSIS-Psy score along with 95 % confidence intervals and exact *p* value. We will investigate whether further adjustment for any variables exhibiting marked imbalance at baseline influences the primary findings.

Earlier effects on the primary outcome will be investigated in a secondary analysis by comparing the arms at 6 months after randomisation. Similar analyses using appropriate regression models depending on outcome type will be conducted for secondary outcomes. Additional, secondary analyses of the primary outcome will take three general forms. First, the influence of missing data will be investigated using sensitivity analyses. Second, the effect of adherence to treatment will be investigated using allocation respecting methods such as complier averaged causal effects modelling using instrumental variable regression. Third, appropriate interaction terms will be entered into the primary regression analyses for MSIS-Psy in order to conduct pre-specified subgroup analyses according to MS type, baseline MSNQ, and baseline Doors and People scores. Since the trial is powered to detect overall differences between the groups rather than interactions of this kind, the results of these exploratory analyses will be presented using confidence intervals and interpreted with due caution.

### Health economic evaluation

The cost-effectiveness will be assessed from the perspective of the UK National Health Service and personal social services. The costs associated with the intervention will be determined by calculating the cost of staff time, materials and travel costs for providing the intervention. These will be compared with changes in the number of visits to General Practitioners, hospital, prescribed medication, and social services contacts in the intervention and control groups during the investigation. The costs will be compared with the outcomes generated and a series of incremental cost-effectiveness ratios computed, including a cost/quality-adjusted life year analysis, based on changes in EQ-5D. A series of one-way sensitivity analyses will be undertaken to determine the extent to which baseline findings change in light of parameter variation. Given the limited time duration of the study and follow-up, a decision analytic model will be constructed to determine the cost-effectiveness of the intervention from a lifetime perspective. A series of scenarios will be constructed to reflect the extent to which differential outcomes can be predicted to continue over longer time periods, using expert opinion and information available in the literature. A probabilistic sensitivity analysis will be carried out to determine the extent to which the intervention can be regarded as representing value for money.

### Assessment of safety and adverse events

The risks of taking part in the study have been assessed as low. There are, however, non-specific risks for participants involved in travelling to the research sites. Also participants may experience some distress if they find they are not performing as well as they think they should on cognitive assessments. However, distress caused in this way is considered very unlikely, and any distress caused is likely to be mild. This distress will be managed by the assistant psychologists who will be qualified to deal with such situations, and make necessary referrals to the participant’s General Practitioner, if needed. For the intervention group, this will also be dealt with during the course of the intervention. As the risk overall has been assessed as low, no adverse events or serious adverse events will be reported for this study. However, as a safety outcome, the number of participants who show an increase in scores on the GHQ30 greater than 30 points between baseline and 6-month assessment will be monitored by the Data Monitoring Committee. In addition, adverse outcomes, such as hospitalisation and distress, will be recorded.

### Participants who withdraw

No withdrawal criteria have been specified, and participants have the right to withdraw from the study at any time. If possible, the reasons for leaving the study will be recorded, but participants are not obliged to give reasons. Participants will be assured that withdrawal will not affect the care they receive. They will be informed at the start of the study that data collected up to the point of withdrawal will be retained and may be used in the final analysis. There will be no replacement of participants who withdraw.

All reasonable attempts will be made to contact any participant lost to follow-up during the course of the study in order to complete assessments.

### Feedback interviews

A feedback interview will be conducted between the 6- and 12-month appointments, with 32 purposefully selected and willing participants, 16 from each group. This will include four intervention and four control participants from each participating centre. The ‘maximum-variation’ selection strategy will be designed to include participants with varying levels of memory impairments, and with varying social circumstances. The interviews will be conducted by a PhD student who will not be involved with the participants’ treatment or outcome assessment, thereby reducing social desirability response bias. The PhD student will become aware of the group allocations during the interview so will not be blind to the intervention. The interview will be audio recorded using a digital recorder, transcribed, and analysed using a thematic analysis, following the protocol prescribed by Braun and Clarke [39]. Participant consent for the interviews will be sought separately. The interviews will provide important feedback on participants’ perception of their progress and, for those in the intervention groups, the quality of the intervention provided, and will serve as a process measure. Insights from this qualitative analysis will serve to inform developments of the intervention programme in the future and to generate user-oriented proposals about areas for further investigations. For those in the control group the interviews will provide confirmation of the nature of usual care received.

### Criteria for terminating the study

The study maybe stopped as a whole because of a change in opinion of the Research Ethics Committee, safety concerns or issues with study conduct at the discretion of the sponsor.

### Trial management

A Trial Management Group (TMG) will be convened and meet regularly. This group will be in charge of the everyday running of the trial. A Trial Steering Committee (TSC) will oversee the conduct of the study and will have an independent chair. Two service user representatives will be members of this group who will advise on recruitment strategies, monitor progress with recruitment, and check adherence to the study protocol. Observers from the National Institute for Health Research, Health Technology Assessment programme (the funder) will be invited to TSC meetings. The Data Monitoring Committee will be an independent group, the members of which have no other involvement with the study. Members of this committee will include rehabilitation professionals and an experienced study statistician. It will safeguard the interests of trial participants, with particular reference to safety and the efficacy of the intervention, monitor the overall progress and conduct of the trial and assist and advise the investigators so as to protect the validity and credibility of the trial.

### Service user involvement

Our service user representative (MM) has MS and cognitive problems. Her role has been to advise on recruitment and dissemination options, and she will contribute to the lay summary of the project. This service user will sit on the TMG. Two other service user representatives will be recruited to the TSC from the MS Society. Another service user will review participant information sheets and any documents that will be read by participants. They will also contribute to dissemination to service users. Service users will contribute to project management decisions, recruitment, consent (the development of participant information sheets), data gathering (through developing participant information leaflets explaining the study), interpretation of findings (through the development of recommendations for practice and patient information leaflets about therapy), and dissemination of the findings through existing networks.

### Definition of a protocol deviation

A protocol deviation is an unanticipated or unintentional divergence or departure from the expected conduct of a study inconsistent with the protocol, consent document or other study procedures. All protocol deviations will be recorded on the electronic case report form by local investigator staff.

## Discussion

This study was conceptualised in response to a commissioned call for studies of cognitive rehabilitation for people with MS. Based on our pilot work [14] and feedback from participants [15] we decided to have a usual care control group, as having a self-help control group was difficult to organise and problematic to facilitate.

Our choice of primary outcome measure was based on the need to consider the impact of cognitive problems on everyday life. We have retained both objective measures of attention and memory and self-reported measures, but these are secondary outcomes. Most objective measures have poor ecological validity.

The content of the intervention is based on a treatment manual, which includes the description of each session and suggested homework tasks. The design was pragmatic and reflects a balance between what it is desirable to cover in terms of teaching people strategies to cope with memory problems, yet at the same time being of sufficiently low intensity that it could be delivered in clinical services for people with MS if found to be effective. The sessions include discussion, as it is hoped that participants will learn from the experience of others. Therefore, it will not be possible to document in detail the exact content of the intervention and time spent on individual activities. However, the intervention is designed to be individualised according to the participants’ cognitive problems and reflects clinical practice, which is appropriate for a pragmatic trial. There is also video recording of a sample of sessions so that it will be possible to check that the intervention was delivered according to the manual.

We anticipate that one of the biggest challenges to recruitment to this study will be potential participants’ own memory problems. Even those individuals interested in taking part in this trial may forget to respond to our invitation letter. To address this, we have ethics approval to include a single telephone call to follow-up non-responders to enquire whether they remember receiving the letter and whether they would like to participate.

## Trial status

The first centre was open to recruitment on 1 March 2015 and the first participant consented on 20 March 2015. At the time of resubmission, 80 people have consented and 39 have been randomised. Recruitment is due to finish on 28 February 2017.

## Abbreviations

BRBN: Brief Repeatable Battery of Neuropsychological Tests
EMQ: Everyday Memory Questionnaire
EQ-5D-5L: Euroquol five dimensions five levels
FSS: Fatigue Severity Scale
GHQ30: General Health Questionnaire 30 item
GNDS: Guys Neurological Disability Scale
ICC: Intra-cluster correlation coefficient
MS: Multiple sclerosis
MSIS: Multiple Sclerosis Impact Scale
MSIS-Phys: Multiple Sclerosis Impact Scale Physical Subscale
MSIS-Psy: Multiple Sclerosis Impact Scale Psychological Subscale
MSNQ: Multiple Sclerosis Neuropsychological Screening Questionnaire
NCTU: Nottingham Clinical Trials Unit
RA: Research assistant
RCT: Randomised controlled trial
TMG: Trial management group
TSC: Trial steering committee
